# Reduced stress responsiveness in pregnancy: Relationship with pattern of forebrain c-*fos* mRNA expression

**DOI:** 10.1016/j.brainres.2010.08.041

**Published:** 2010-10-28

**Authors:** Richard J. Windle, Susan A. Wood, Yvonne M. Kershaw, Stafford L. Lightman, Colin D. Ingram

**Affiliations:** a University Research Centre for Neuroendocrinology, University of Bristol, Bristol Royal Infirmary, Bristol, BS2 8HW, UK; b School of Nursing, Midwifery and Physiotherapy, University of Nottingham, Queen's Medical Centre, Nottingham NG7 2UH, UK; c Institute of Neuroscience, Newcastle University, Medical School, Framlington Place, Newcastle upon Tyne NE2 4HH, UK

**Keywords:** ACTH, Gestation, Progesterone, Immediate early gene expression, PVN

## Abstract

Stress-induced activation of the hypothalamo-pituitary-adrenal (HPA) axis is known to be attenuated during late pregnancy and throughout lactation. To investigate the neural basis of this stress hyporesponsiveness we examined the changes in the restraint-induced HPA response and accompanying forebrain expression of c-*fos* mRNA that occur in rats between days 16 (D16) and 19 (D19) of gestation, times associated with declining levels of progesterone, a potential mediating factor. Compared to D16, the D19 group showed a significantly attenuated release of ACTH following 30 min restraint. This reduced HPA response was accompanied by significantly lower levels of restraint-induced c-*fos* mRNA expression in the hypothalamic paraventricular nucleus. Other areas of the forebrain, including medial amygdala, piriform cortex, and ventrolateral septum, showed low c-*fos* mRNA expression in non-stressed (control) animals and a large increase following restraint, the magnitude of which was similar between D16 and D19 animals indicating no involvement in the differential HPA response to stress. However, a markedly different pattern of c-*fos* mRNA expression was observed in other brain areas, including barrel cortex and CA1 ventral and CA3 regions of the ventral hippocampus: D19 animals had low control expression which was increased by restraint, but D16 control animals had raised c-*fos* mRNA expression which was not further elevated by stress. These data demonstrate that region-specific changes in basal and stress-induced cellular activity occur during a period of late gestation coincident with attenuated HPA responsiveness. These changes in neuronal activity may contribute to the adaptive processes that prepare the mother for parturition and lactation.

## Research Highlights

►Differential patterns of neuronal activation accompany stress in pregnancy. ►PVN shows reduced stress activation in late pregnancy matching reduced ACTH levels. ►Most other forebrain areas show similar stress activation in mid and late pregnancy. ►Hippocampus and cortex show high basal, but no stress activation in mid pregnancy. ►Specific changes in forebrain activity underlie maternal neuroendocrine adaptation.

## 1 Introduction

Pregnancy and the time around parturition are associated with profound behavioural and physiological adaptations in the mother which ensure reproductive success (Russell et al., 2001; Brunton and Russell, 2008). One of the most well characterised of these adaptive changes is the marked attenuation of the hypothalamo-pituitary-adrenal (HPA) response to physical or psychological stress, which starts during pregnancy and lasts throughout lactation (Stern et al., 1973; Lightman and Young, 1989; Windle et al., 1997a; Neumann et al., 1998; Johnstone et al., 2000; Lightman et al., 2001; Douglas et al., 2003; Brunton et al., 2008). In addition to this neuroendocrine adaptation, anxiety-related behaviour and emotional responsiveness to stressful stimuli are reduced in late pregnant and lactating rats. These adaptations are particularly important for the promotion of postnatal maternal behaviour and for prevention of exposure of the offspring to excessive levels of glucocorticoid hormones (Slattery and Neumann, 2007; Brunton and Russell, 2008).

Although much work has been done on characterising the established changes in stress responsiveness at the time of parturition (day 21 (D21) of gestation in the rat) and into lactation, little is known about the adaptive mechanisms that are happening earlier in pregnancy during the onset of hyporesponsiveness. We first reported that for animals tested on D19–21 of pregnancy the levels of corticosterone induced by 30 min restraint stress were significantly attenuated compared to virgin controls (da Costa et al., 1996). Furthermore, the corresponding level of c-*fos* mRNA expression in the hypothalamic paraventricular nucleus (PVN) indicated that central activation of the HPA axis was compromised in these late pregnant animals. Rats at D19 of pregnancy also show a reduced ACTH response to forced swim stress (Johnstone et al., 2000) and mice on D17.5–18.5 of gestation, 1 day before the expected day of delivery, show an attenuated ACTH response to a novel environmental stress which is accompanied by a complete suppression of the induction of the immediate-early gene *nur77* in the PVN (Douglas et al., 2003). Neumann et al. (1998) reported that the psychological stress of combined exposure to the elevated plus maze and forced swim evoked an increase in ACTH in virgin animals and rats at D10 of gestation, but this response was attenuated later in pregnancy (D15, D18 or D21). They concluded that this was partly an effect of a change in pituitary responsiveness as the ACTH response to i.v. corticotrophin-releasing hormone (CRH) declined between D11 and D16. Finally, in determining the timing of the onset of this hyporesponsiveness we have shown that a marked change in stress responsiveness occurs between D16 and D19: at D16 basal corticosterone levels were elevated and a 10-min period of white noise stress induced a further rise in corticosterone, but by D19 this stress response was absent (Lightman et al., 2001).

One of the factors believed to orchestrate this stress hyporesponsiveness is the marked switch in progesterone and estrogen levels that occurs following regression of the corpus luteum (luteolysis). This results in a decline in levels of progesterone between D15/D16 and D19/D20 (Garland et al., 1987), which is believed to trigger a number of the prepartum adaptive changes (Russell et al., 2001). We have demonstrated the importance of such steroid changes for stress responsiveness by showing that a steroid model of luteolysis in non-pregnant animals causes a significant decrease in stress-induced HPA activation (Windle et al., 2006).

In order to further investigate the onset of stress hyporesponsiveness in pregnancy we have compared the HPA response to restraint stress on D16 and D19 and have examined the associated changes in stress-induced neural activity by analysis of the forebrain expression of the immediate-early gene *c-fos* which has been used previously to map the stress responsive circuitry activated by restraint (Cullinan et al., 1995; da Costa et al., 1996; Dayas et al., 2001; Windle et al., 2004).

## 2 Results

### 2.1 HPA response to restraint stress

**Fig. 1:**
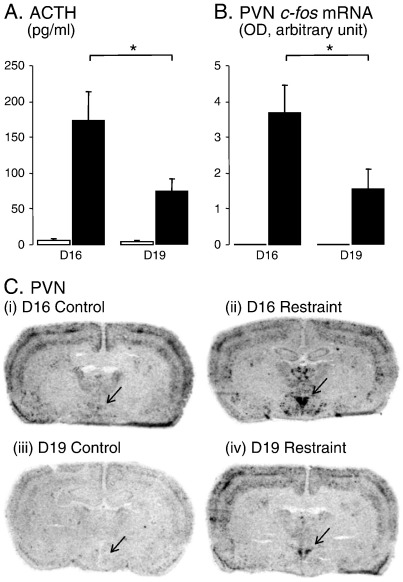
HPA and PVN activation by restraint stress. (A) Mean (± SEM) plasma ACTH concentrations in control animals (open bars) or following a 30 min period of restraint (closed bars) at day 16 (D16) and day 19 (D19) of gestation. (B) Mean (± SE, *n* = 7–8) integrated optical density (OD) measurements of c-*fos* mRNA expression in the PVN of control animals (open bars) and animals following a 30 min restraint (closed bars). *significantly different by *post-hoc* Tukey's test, *P* < 0.05. (C) Representative autoradiograms showing the hybridized c-*fos* mRNA signal throughout frontal sections at the level of the PVN from non-restrained (i) and restrained (ii) rats at day 16 of pregnancy, and from non-restrained (iii) and restrained (iv) rats at day 19 of pregnancy. Arrows indicate the positions of the PVN. Note that in all cases the presence of the PVN was confirmed after staining with cresyl violet.

Restraint stress caused a significant increase in plasma ACTH levels in both the D16 and D19 groups (*P* < 0.001, *F*_1,35_ = 30.5), however, the response was significantly smaller at D19 (*P* < 0.05, *F*_1,35_ = 4.6; Fig. 1A). Consistent with these changes in HPA activity, *c-fos* mRNA levels in the PVN were very low or undetectable in the control, non-stressed animals (Fig. 1C) and, whilst restraint caused a marked increase in both groups (*P* < 0.001, *F*_1,27_ = 31.6, Fig. 1C, Table 1), the magnitude of response was significantly attenuated in the D19 group (Fig. 1B) as shown by a significant interaction between stage of pregnancy and stress (Table 1).

### 2.2 Forebrain neuronal activation in unstressed animals

As in previous studies, control (unstressed) animals from D19 showed very low expression of *c-fos* mRNA throughout the forebrain (Figs. 1Ciii; 2Aiii; Biii). However, a striking difference was seen in D16 animals in that there was a high level of expression in several areas in the absence of any applied stress. A two-way ANOVA between pregnancy stage and all 16 forebrain areas analysed revealed this effect to be highly significant (*P* < 0.001, *F*_(1,195)_ = 31.3). This difference in expression was particularly notable across the pyramidal layers of the neocortex (especially in barrel cortex) and in subfields CA1 ventral and CA3 of the ventral hippocampus (Figs. 1Ci; 2Bi; Table 1). Higher basal *c-fos* mRNA expression at D16 was also seen in other subfields of the hippocampus, in the dorsal endopiriform cortex, and in various divisions of the thalamus (anterodorsal, anteroventral and paraventricular nuclei) (Figs. 1 Ci; 2Ai; Bi), although in these areas variance in the expression levels meant that the difference between groups did not reach statistical significance (e.g. CA2, Fig. 3C; Table 1). This higher expression appeared to be specific and confined to particular areas of the forebrain. Other areas measured, such as the piriform cortex, ventrolateral septum, medial amygdala (MeA), and principal nucleus of the BST, showed no such difference in basal expression between D16 and D19 (Fig. 4).

### 2.3 Forebrain neuronal activation following restraint stress

**Fig. 2:**
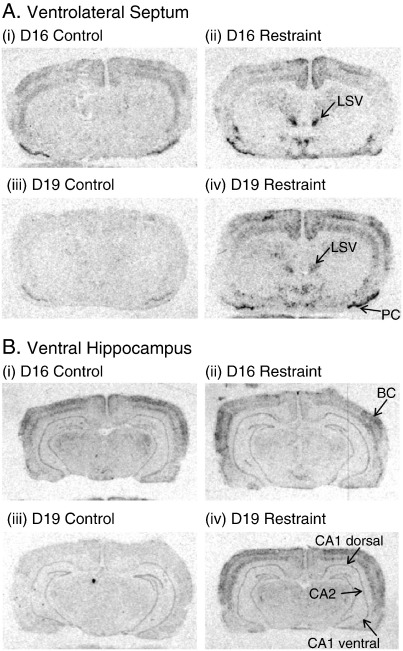
Autoradiograms showing the hybridized c-*fos* mRNA signal throughout forebrain sections at the level of the ventrolateral septum (LSV) (A) and ventral hippocampus (B). Sections are from non-restrained (i) and restrained (ii) rats at day 16 (D16) of pregnancy and from non-restrained (iii) and restrained (iv) rats at day 19 (D19) of pregnancy. BC, barrel cortex, PC, piriform cortex.

**Fig. 3:**
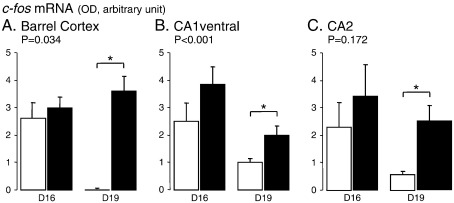
Optical density (OD) measurements of *c-fos* mRNA expression within layer V of the cortex (barrel cortex, A), and the CA1 ventral (B) and CA2 (C) subfields of the ventral hippocampus of control animals (open bars) or animals subjected to a 30-min period of restraint (closed bars) on day 16 (D16) or day 19 (D19) of gestation. Values are the mean ± SE of integrated optical density measurements (*n* = 7–8). *Significantly different by *t*-test, *P* < 0.05. Statistical values show the effect of stage of pregnancy (two-way ANOVA).

**Fig. 4:**
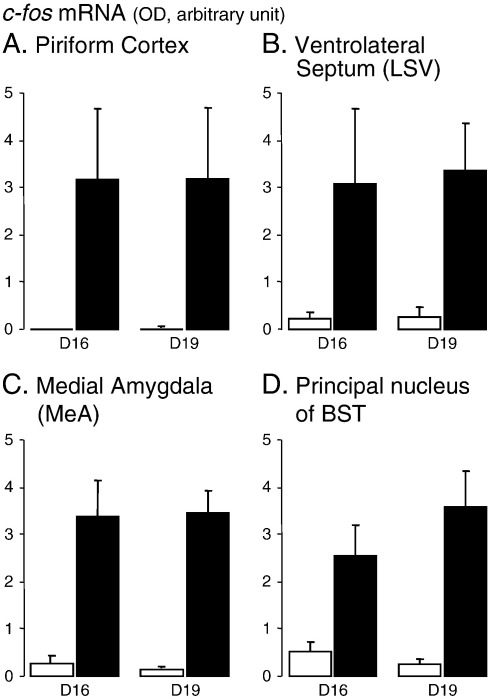
Optical density (OD) measurements of *c-fos* mRNA expression within the piriform cortex (A), ventrolateral septum (B), medial amygdala (C), and principal nucleus of the bed nucleus of the stria terminalis (D) of control animals (open bars) or animals subjected to a 30-min period of restraint (closed bars) on day 16 (D16) or day 19 (D19) of gestation. Values are the mean **±** SE of integrated optical density measurements (*n* = 5–8).

As expected, the application of restraint stress significantly increased *c-fos* mRNA expression in a number of areas (Table 1). In several areas which displayed low basal gene expression at both D16 and D19, stress led to an increase in *c-fos* mRNA, the magnitude of which was the same at the two stages of gestation (Fig. 4). This can be seen for the ventrolateral septum (LSV; Figs. 2A; 4B), MeA, principal nucleus of the bed nuclei of the stria terminalis, and piriform cortex. In contrast, for those areas which displayed elevated basal expression at D16, stress caused a significant increase in the D19 animals (Fig. 3), but had no significant effect on the already elevated levels at D16.

## 3 Discussion

### 3.1 Attenuation of stress-induced HPA activation

The present data provide evidence of an increasing attenuation of stress-induced HPA activation between D16 and D19 of pregnancy. Consistent with our data using the more mild stress of white noise (Lightman et al., 2001), the restraint-induced levels of ACTH were significantly lower at D19 compared to D16. It has been proposed that this pregnancy-related stress hyporesponsiveness may arise from reduced pituitary response to CRH (Neumann et al., 1998), and Johnstone et al. (2000) have shown that basal expression of CRH mRNA in the PVN declines between D16 and D21 of pregnancy which might contribute to reduced pituitary drive. Whilst changes in pituitary drive between D16 and D19 may partly account for the reduced HPA response, the present data clearly show that there is also a marked reduction in stress-induced activation of the PVN. This pregnancy-related attenuation of PVN activation is consistent with data comparing virgin and late pregnant mice showing reduced stress-induced *nur77* mRNA expression (Douglas et al., 2003). However, our data also clearly show that this reduced afferent (feed-forward) activation of the PVN involves a central mechanism and is not due to differences in either the sensory or cognitive perception of restraint stress, since there were no differences in the magnitude of the *c-fos* mRNA response in many other limbic and cortical regions. Furthermore, measurements made across 16 stress-sensitive regions of the forebrain suggest that this attenuated activation may be highly localised to the PVN.

Importantly, the present study focussed on the adaptive changes in HPA activation which are occurring during late pregnancy. We have not made comparisons against non-pregnant (virgin) animals as we were interested primarily in the potential hyporesponsivity occurring as a result of the steroid changes known to occur over this period of pregnancy in the rat (Crowley et al., 1995; Windle et al., 2004). Therefore the term “hyporesponsive” as used here is relative between the two pregnant groups and the timing within pregnancy. Although the ACTH levels measured here were not compared with a virgin group, the response seen at D16 was comparable in magnitude to that we have previously reported in similar animals undergoing another form of mild psychological stress, noise stress (Windle et al., 1997a). Similar declines in the ACTH response to mild stresses has been reported between virgin and pregnant animals after day 20 of pregnancy following exposure to the elevated plus maze ( Neumann et al., 1999), air puff (Neumann et al., 2003) as well as interleukin1-β challenge (Brunton et al., 2009). Comparison of the patterns of *c-fos* mRNA expression with non-pregnant animals is also difficult, given the nature of the analysis which provides comparative units in arbitrary values. This is compounded by the fact that few studies have measured *c-fos* mRNA expression patterns in female animals, the fact that many studies report stressed, but not control values, and also the fact that *c-fos* mRNA expression within the forebrain has been shown to vary with the stage of the oestrous cycle. (Figueiredo et al., 2002). However, we have previously reported *c-fos* mRNA expression patterns to a comparable restraint stress in female rats given an ovarian steroid regime to mimic the normal diestrus range (Windle et al., 2004). In these animals similar patterns of *c-fos* mRNA expression to the D16 animals were seen within the PVN, piriform cortex, LSV and medial amygdala. However, the high levels of basal expression reported here in D16 animals in areas such as subfields of the hippocampus were not seen in those animals, suggesting that this may be specific to this stage of pregnancy. Despite these comparisons, we cannot determine whether the HPA and/or *c-fos* mRNA responses at D16 are already attenuated with respect to the virgin state and only continue to decline towards D19 (as might be suggested from ACTH responses to combined elevated plus maze and forced swim (Neumann et al. (1998)), or whether the onset of hyporesponsiveness occurs over this time frame. Nevertheless, the more than 50% reduction in restraint-induced ACTH and PVN *c-fos* mRNA demonstrates that important adaptive changes are operating over this time.

Immediate-early gene mapping studies have indicated that the LSV and MeA are key areas gating afferent activation of the PVN and which contribute to adaptive changes in PVN activation and HPA responsiveness. During early lactation (days 3–4 post-partum) both these areas show highly attenuated restraint-induced activation and the PVN response is completely abolished (da Costa et al., 1996). However, the present data show that the attenuation of the HPA axis and the reduced activation of the PVN which is happening between D16 and D19 of pregnancy occur without changes in either LSV or MeA, suggesting that different mechanisms may operate at different times in the reproductive cycle. It is possible that the hyporesponsiveness during pregnancy may involve mechanisms that reside solely at the level of the PVN/HPA axis, whilst after parturition an alternative, or additional, mechanism may involve higher limbic circuitry (da Costa et al., 1996).

### 3.2 Potential regulatory mechanisms

One factor which has been proposed to underlie the stress hyporesponsiveness in lactation is the neuropeptide oxytocin, expression of which and that of its receptor increases around parturition (Insel, 1990; Ingram and Wakerley, 1993; Lightman et al., 2001). We have previously shown that i.c.v. infusion of oxytocin to steroid-treated, non-pregnant rats will attenuate the HPA response to both noise (Windle et al., 1997b) and restraint (Windle et al., 2004), and mapping of *c-fos* mRNA showed that oxytocin suppressed gene expression in the PVN, LSV and all subfields of the dorsal hippocampus (Windle et al., 2004). However, the lack of any differences in stress-induced *c-fos* mRNA in the LSV and dorsal hippocampus in the current study suggests that a different mechanism is operating during pregnancy, and this is consistent with data showing that an oxytocin receptor antagonist cannot reverse the stress hyporesponsiveness at parturition (Neumann et al., 2003).

An alternative mechanism which may be operating during pregnancy is a change in afferent noradrenergic drive. It has recently been proposed that pregnancy-related reduction in HPA activation is due to suppression of PVN noradrenaline (NA) release caused by increased pre-synaptic opioid inhibition (Brunton et al., 2008; Russell et al., 2008). It has been shown that, compared to the normally robust response in virgin animals, during late pregnancy (D21) NA release into the PVN measured during 10 min swim stress is completely lost and the level of α_1A_-adrenergic receptor mRNA in the PVN is significantly reduced (Douglas et al., 2005). These authors suggested that this change in NA drive contributed to the reduced HPA response. However, ACTH levels did increase significantly in both virgin and D21 animals in response to 60 s swim stress, suggesting PVN NA was not obligatory. Using measures of transmitter levels in the hypothalamus of D15 and D20 pregnant rats, Glaser et al. (1992) also detected a significant decrease in NA levels and corresponding rise in MHPG/NA ratio (usually taken as an index of increased turnover). More recently Macbeth et al. (2008) saw a similar pattern of changes in NA and MHPG/NA ratio in both hypothalamus (medial preoptic area) and prefrontal cortex when comparing D9 and D18 pregnant animals. Interestingly, measurements in the CA1 hippocampus showed the converse pattern–a four-fold increase in NA levels and halving of the MHPG/NA ratio–a result not detected by Glaser et al. (1992) using measurements of the whole hippocampus. Thus, if altered NA drive to the PVN does underlie the pregnancy-related hyporesponsiveness then it is necessary to explain these regional differences in release and metabolism, and why areas like the BST which receive NA afferents in common with the PVN (Woulfe et al., 1988; Pacak et al., 1995; Gaykema et al., 2007) do not show similarly reduced *c-fos* activation.

### 3.3 Changes in basal c-fos mRNA expression

One unexpected finding in this study was the higher cortical and hippocampal expression of *c-fos* mRNA at D16 compared to D19. Despite the fact that these levels were equivalent to those achieved by 30-min restraint and were not further increased by this stress, we believe this elevated expression was not because for some reason this group of animals were stressed. This is evinced by the fact that many stress-sensitive regions (e.g. PVN, LSV, MeA, BST, piriform cortex) showed no similarly elevated *c-fos* expression (e.g. Figs. 1C and 2A), and by the fact that ACTH and corticosterone levels were no different between control (non-stressed) animals at D16 and D19. Therefore, we believe that this pattern of expression relates to a physiological change in cortical and hippocampal function occurring over this period.

Changes in “basal” *c-fos* expression has not been previously noted in pregnancy. However, as our study only compared the changes occurring during pregnancy it is not possible to conclude which pattern of expression reflects the non-pregnant state. Lin et al. (1995) reported no notable basal expression of Fos protein in the supraoptic nuclei and PVN of pregnant rats at D10 and D20, although this did dramatically increase at parturition. Furthermore, analysis of Fos-immunoreactive cells across a wide number of brain regions showed that expression was low at D21 of gestation but increased once parturition commenced (Lin et al., 1998). This might suggest that there is widespread suppression of Fos expression in the period leading up to parturition.

Beyond their usefulness as markers of neuronal activation, one of the physiological roles for fos-related genes is regulation of maternal behaviour and deletion of the fos-B gene has been shown to cause a major deficit in nursing behaviour (Brown et al., 1996; Kuroda et al., 2008). It is possible that the decline in cortical and hippocampal *c-fos* mRNA between D16 and D19 relates to changes in maternal responding, and has no relevance to these areas being also involved in the stress response. A trigger for these changes may be the significant decline in progesterone levels in these animals (see Section 4.4) which is likely to arise from the luteolysis. Since GABA inhibition is known to be sensitive to the changing ovarian steroid levels in the prepartum period (Biggio et al., 2009), it is possible that the changing expression of fos at D16 and D19 may reflect an underlying GABAergic regulation of maternal reactivity in the pregnant rat as it does later in lactation (Lee and Gammie, 2007).

## 4 Experimental procedures

### 4.1 Animals

Female Sprague–Dawley rats were obtained from Bantin and Kingman (Hull, UK). Animals had free access to food and water throughout the study and were maintained on a 14 h light: 10 h dark illumination schedule (lights on at 05.00). Animals were mated and the timing of pregnancy confirmed by presence of vaginal plugs and subsequent gestation. Animals remained group-housed until 7 days prior to study after which they were singly housed. All experiments were carried out in accordance with the European Communities Council Directive 86/609/EEC and were designed to minimize pain and discomfort.

### 4.2 Effect of restraint on HPA activity

Rats at D16 and D19 of pregnancy were removed from their home cage between 9.00 and 11.00 h and placed in a Perspex tube of suitable diameter for 30 min. The diameter was selected to prevent the animal turning around but to still allow space for limited movement (internal diameter 6–8 cm). After 30 min animals were killed by decapitation. Trunk blood was collected into tubes containing 10 μl saturated EDTA for ACTH determination. Tubes were centrifuged and the plasma collected for subsequent hormone analysis. Brains were removed rapidly and frozen on dry ice for analysis of *c-fos* mRNA expression. Blood and tissues were collected from non-restrained animals at equivalent times.

### 4.3 c-fos mRNA determination

All brains were stored at −80 °C prior to analysis. Cryostat sections (12 μm) were cut from forebrain regions known to exhibit increased *c-fos* mRNA expression following restraint (Cullinan et al., 1995; da Costa et al., 1996). Collection of sections onto gelatin-coated slides commenced rostral to the anterior nuclei of the BST (bregma + 0.2 mm, Paxinos and Watson, 1998) and continued to a level that was caudal to the central amygdala (bregma −3.3 mm). A block of tissue approximately 1 mm thick was then discarded before a final series of sections were collected through the region of the ventral hippocampus (starting at bregma −4.8 mm). A collection regime was adopted that resulted in each slide containing four sections each separated by approximately 50 μm through the region of interest. Thus, each slide contained a representative cross-section from a 200 μm thick block through the region to be studied. All sections were stored at −80 °C until analysis.

*In situ* hybridization for *c-fos* mRNA was carried out as follows; sense and antisense rat *c-fos* transcripts incorporating ^35^S-UTP were generated from the vector pGEM-3Z by inserting a 680 bp fragment of rat *c-fos*. The probes were transcribed using an Sp6/T7 transcription kit according to the manufacturer's instructions (Roche Diagnostics Ltd., Lewes, UK). Tissue sections were fixed in 4% paraformaldehyde and hybridized overnight at 50 °C in hybridization buffer containing 50% formamide, 4 × saline sodium citrate (SSC), 1× Denhardt's solution (0.02% Ficoll, 0.02% polyvinylpyrrolidone, 0.02% BSA), 10% dextran sulphate (mol. wt. 500,000) and 10^6^ cpm/slide. Before adding riboprobe to the hybridization buffer, it was mixed with 2 μl of nucleic acid solution/slide (500 μg/ml sheared, single stranded salmon testis DNA and 250 μg/ml yeast tRNA), heated to 65 °C for 5 min and quenched on ice. Following hybridization, coverslips were gently lifted off in 1× SSC at room temperature and the slides washed for 15 min in two changes of 1× SSC/50% formamide at 50 °C. Sections were then rinsed briefly in 1× SSC at 37 °C and incubated in 1× SSC containing 20 μg/ml RNase A for 30 min at 37 °C. Sections were again rinsed in 1× SSC, then washed in 3 × 15 min changes of 1 × SSC/50% formamide at 50 °C, followed by two room temperature washes in 1× SSC for 5 min each. Slides were briefly dipped in water then air-dried.

Five separate brain levels were analyzed and sections for a given level of the brain were hybridized in the same reaction and exposed to photographic film (Hyperfilm, Amersham, Bucks, UK) together with a series of ^35^S standards. Exposure times were 21 days except for the PVN which was 11 days to ensure that the high expression in stressed animals did not saturate the film. Following hybridization each slide was stained with cresyl violet in order to confirm the presence of the structures of interest and any case where the structure was absent or could not be accurately quantified was excluded from further analysis. Numbers of replicates in each treatment group are indicated in the figure legends. All analysis and any exclusions were conducted blind to the treatment groups. The developed photographic films were subject to densitometric analysis using public access software “Image” (http://rsb.info.nih.gov/nih-image). The integrated optical density (area detected above threshold x mean optical density within the thresholded area) was measured for each of the structures expressing *c-fos* message in the sections; the mean value for a given animal being determined from all sections containing that structure. Day of pregnancy and stress procedures were unknown to those performing the *in situ* analysis.

### 4.4 Hormone determination

ACTH levels were determined using a commercially available immunoradiometric assay kit (Nicholls Institute Diagnostics, Capistrano, CA, USA). Plasma ovarian steroid levels in these animals have been previously reported (Windle et al., 2006) and showed a significant decline in progesterone (D16: 59.6 ± 11.9 ng/ml (*n* = 7) vs. D19: 34.5 ± 5.2 ng/ml (*n* = 6), *P* < 0.05) but no significant change in estradiol-17β (D16: 132.7 ± 14.0 ng/ml (*n* = 8) *vs*. D19: 113.7 ± 9.3 ng/ml (*n* = 12)).

### 4.5 Statistical analysis

Values represent either the mean ± SE for groups. Absolute measurements for hormone concentrations are given, but gene expression data is expressed in arbitrary optical density units. Primary analysis involved a two-way ANOVA to determine significant effects of stress and stage of pregnancy on the hormone and gene expression data. Analysis was performed for within-region differences in expression and statistical comparisons between areas were not undertaken. Values of *P* < 0.05 are reported as significant.

## Figures and Tables

**Table 1:** *P*-values from the statistical analysis of the effects of restraint and the stage of pregnancy on *c-fos* mRNA expression in areas of the forebrain (values shown are derived from two-way ANOVA). NS = *P* > 0.05.

	Restraint	Stage of Pregnancy	Interaction
*Telencephalon*			
Neocortex (layer 3 of barrel cortex)	0.002 (*F*_1,25_ = 12.1)	0.034 (*F*_1,27_ = 5.0)	0.007 (*F*_1,27_ = 8.4)
Piriform cortex	< 0.001 (*F*_1,26_ = 24.5)	NS	NS
Dorsal endopiriform n.	< 0.001 (*F*_1,26_ = 28.9)	NS	NS
Medial amygdala	< 0.001 (*F*_1,27_ = 51.9)	NS	NS
BST—Principal n.	0.003 (*F*_1,26_ = 10.5)	NS	NS
LSV	< 0.001 (*F*_1,25_ = 22.5)	NS	NS

*Ventral hippocampus*			
CA1 dorsal	0.030 (*F*_1,27_ = 5.2)	NS	NS
CA1 ventral	0.015 (*F*_1,27_ = 6.7)	< 0.001 (*F*_1,27_ = 13.6)	NS
CA2	0.029 (*F*_1,27_ = 5.3)	NS	NS
CA3	NS	0.012 (*F*_1,27_ = 7.3)	NS
CA4	NS	NS	NS
Dentate gyrus	NS	NS	NS

*Diencephalon*			
PVN	< 0.001 (*F*_1,27_ = 31.6)	0.023 (*F*_1,27_ = 5.8)	0.024 (*F*_1,27_ = 5.7)
Anterodorsal thalamus	NS	NS	NS
Anteroventral thalamus	NS	NS	NS
Paraventricular thalamus	0.023 (*F*_1,26_ = 5.9)	NS	NS
